# Patterns of missing data in the use of the endometriosis symptom diary

**DOI:** 10.1186/s12905-018-0578-0

**Published:** 2018-06-08

**Authors:** Christian Seitz, Vivian Lanius, Susanne Lippert, Christoph Gerlinger, Claudia Haberland, Frank Oehmke, Hans-Rudolf Tinneberg

**Affiliations:** 1 Bayer AG, 13353 Berlin, Germany; 2 Department of Obstetrics and Gynecology, University of Saarland, Saarbrücken, Germany; 3 University of Giessen, Giessen, Germany

**Keywords:** Missing data, Electronic patient-reported outcome, Endometriosis

## Abstract

**Background:**

Endometriosis is a common, chronic condition in women of reproductive age that is characterized by the presence of functional endometriotic lesions outside the uterus. The Endometriosis Symptom Diary (ESD) is an electronic patient-reported outcome (ePRO) instrument that assesses women’s experience of endometriosis symptoms, with pain scored using a 0–10 numeric rating scale. This study investigated patterns of data missing from the ESD in the VALEPRO study.

**Methods:**

Post hoc analyses of missing data were conducted.

**Results:**

Of 272 participants using the ESD, 26.5% had no missing diary entries, 46.7% had > 0–5% of entries missing, 13.2% had > 5–10% of entries missing and 13.6% had > 10% of entries missing over the entire study period. The duration of missing episodes (defined as ≥1 consecutive days with missing diary entries) was generally short; most (81.4%) were 1 day. The difference in mean worst pain scores between missing and complete episodes per participant was − 0.1, suggesting that missing episodes were not related to severity of pain. Entries were significantly more likely to be missing on Fridays (18.5%) and Saturdays (22.9%) compared with other days of the week (*p* < 0.0001). Participants in the USA had significantly more long missing episodes than those in Germany (proportions of missing episodes longer than 1 day, 22.6 and 10.5%, respectively; *p* < 0.0001). The proportions of women with ≥1 missing entry were 50.0, 70.2 and 79.8% for women with elementary education, secondary education, and a college or university education, respectively. The proportions of women with ≥1 missing entry were similar for those with and without children (72.2 and 74.3%, respectively).

**Conclusions:**

Most participants were highly compliant with entering data in the ESD and the amount of missing data was low. Entries were significantly more likely to be missing on Fridays and Saturdays compared with other days of the week, and participants in the USA had significantly more long missing episodes than participants in Germany.

**Trial registration:**

Clinicaltrials.gov, NCT01643122, registered 4 July 2012.

## Background

Endometriosis is a chronic, sex hormone-dependent, inflammatory disease that affects approximately 6–10% of women of childbearing age [1, 2]. The disease is characterized by the presence of endometriotic lesions that can develop on the peritoneum, ovaries, fallopian tubes, bladder, ureters or bowel, and can form adhesions between organs [1–4]. The lesions proliferate and haemorrhage in response to hormone level fluctuations during the menstrual cycle, and cause symptoms including pelvic pain, dysmenorrhoea and dyspareunia [2, 5]. These symptoms have a significant impact on health-related quality of life [2, 6, 7].

Patient-reported outcome (PRO) measures provide the most reliable means of assessing the symptoms of endometriosis and their impact on the daily lives of patients. The currently available PRO measures for use in endometriosis [8–11] do not meet the standards defined by the 2009 Food and Drug Administration (FDA) PRO Guidance for Industry [12]. Based on a review of existing literature and extensive qualitative research in women with endometriosis, two new electronic PRO (ePRO) measures have been developed: the Endometriosis Symptom Diary (ESD) and the Endometriosis Impact Scale (EIS). ePROs are typically completed using an electronic hand-held device, and have a range of advantages over conventional paper data collection tools (paper PRO instruments) [13, 14]. They enable real-time data capture, thus providing insight into a patient’s condition between hospital visits. They can also reduce the occurrence of missing data through techniques such as alerts to remind patients to complete the PRO and time-dependent data entry windows to ensure that assessments are completed within the required time frame, leading to more accurate and complete data. In addition, ePRO instruments reduce errors associated with data transfer from filled paper PRO instruments into electronic systems and involve a smaller administrative burden than paper PRO instruments. In summary, ePRO instruments enhance the integrity and accuracy of PRO data captured in clinical trials [13, 14]. The FDA provides guidance to promote the electronic capture of PRO endpoints in clinical trials [12].

The ESD is being developed in close interaction with the FDA, and observations from the present analysis were made during the validation process for the ESD. A non-interventional real-world study has been conducted to investigate the validity of the ESD and EIS (Validation Study for Endometriosis PRO [VALEPRO]; clinicaltrials.gov identifier NCT01643122). VALEPRO has shown strong evidence for the reliability and validity of scores derived from the ESD and EIS [15]. The present article reports the results of post hoc analyses performed to investigate the patterns and potential causes of missing ESD data in the VALEPRO study, with the objectives of examining the level of data missing with the new electronic PRO measure and understanding the causes of missing data in order to minimize them in future studies.

## Methods

### Study design

VALEPRO was a prospective, observational, validation study conducted in the USA and Germany. Of the 272 participants, full data sets from 268 women were analysed in the VALEPRO study (four participants were excluded from the analysis population owing to insufficient information across a number of variables). According to the assessment of the recruiting clinicians using the Clinical Global Impression of Severity scale, the majority of participants were rated as experiencing mild (40.3% [108/268]) or moderate (32.5% [87/268]) endometriosis. In contrast, the participants themselves felt they had more severe endometriosis (as measured using the Patient Global Impression of Severity scale), with the majority reporting moderate (31.0% [83/268]) or severe (26.5% [71/268]) endometriosis.

Participants completed the ESD daily for at least 12 weeks using an electronic hand-held device supplied by the study sponsor. Women whose endometriosis management was changed during the study period were monitored for a further 12 weeks (maximum data collection period of 24 weeks). Missing data were analysed across six 28-day reference periods: 1 (days 1–28); 2 (days 29–56); 3 (days 57–84); 4 (days 85–112); 5 (days 113–140); and 6 (days 141–168). These reference periods were determined at the patient level, using the diary completion dates for each participant. The design of VALEPRO has been reported previously [15].

### Study participants

Women aged 18–45 years were eligible to participate in the study if they had endometriosis confirmed by laparoscopy or laparotomy during the 5 years before the baseline visit and endometriosis symptoms (i.e. pain) during the 4 weeks before the baseline visit, as assessed by the participant using a 0–10 numeric rating scale (NRS), and had otherwise good general health, as evidenced by medical history [16]. Women were excluded from the study for any of the following reasons: presence of diseases or conditions that might have interfered with the conduct of the study or the interpretation of the results; undiagnosed abnormal genital bleeding; abuse of alcohol, drugs or medicine; simultaneous participation in another clinical trial or participation in another clinical trial before study entry that might have had an impact on the study objectives (at the discretion of the investigator); major surgery scheduled for the study period (except therapeutic surgical procedures for endometriosis); close affiliation with the study site; inability to cooperate with the study procedures for any reason; regular use of pain medication owing to other underlying diseases; or known pregnancy [16].

### ESD

**Fig. 1:**
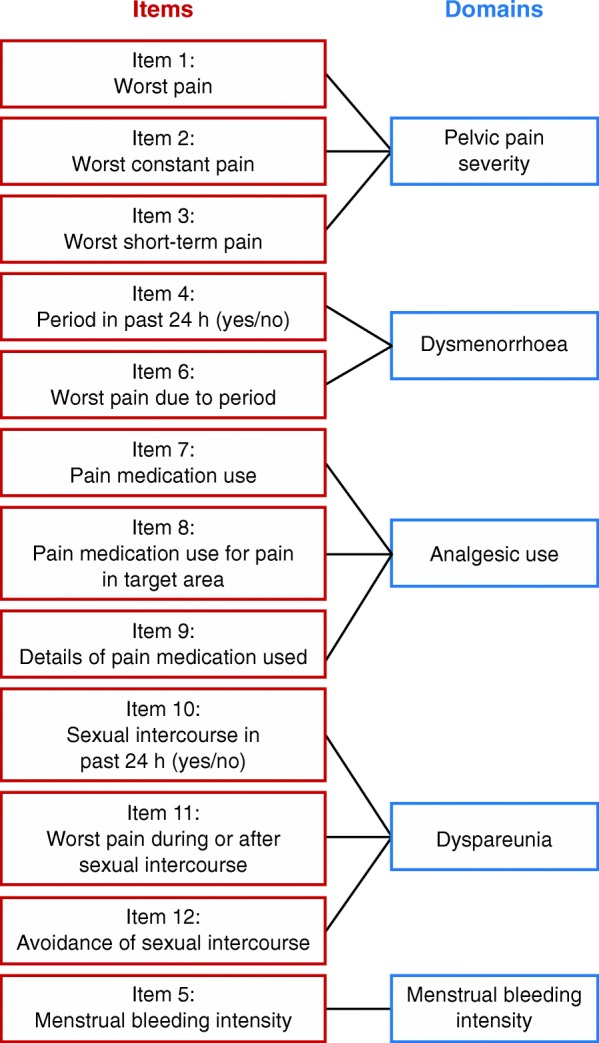
Conceptual framework of the 12-item Endometriosis Symptom Diary version 4.0

The ESD version 4.0 is a 12-item ePRO measure with a 24-h recall period that aims to assess women’s experience of endometriosis symptoms (Fig. 1). Women are instructed to complete the ESD daily between 18:00 h and 00:00 h, and the data entered can be used to derive various scores based on the woman’s assessment of the three main symptoms of endometriosis (pelvic pain, dysmenorrhoea and dyspareunia) at their worst in the preceding 24 h. Symptoms are scored using a NRS of 0–10 where 0 = no pain and 10 = “pain as bad as you can imagine”. Anchoring of pain scores is avoided as far as possible by the ePRO device, preventing the patient from seeing her previous entries. Different aggregated scores over the 28-day period can be calculated from the daily scores (e.g. 28-day-average score or a mean of 7-worst-days-average score).

### Missing data

As a preventative measure, at each hospital visit, participants were reminded to complete their daily diary entries on time, and the ESD was programmed with an alert that notifies participants if they do not complete an entry on time. The ESD presents a logical progression of items, and the technical design is such that the respondent has to answer all questions about these items on any given day within the fixed time window. Therefore, in VALEPRO, it was not possible to have missing data at the item level; only missing data at the form level were possible. Form-level data could be missing because of non-compliance with data entry, a participant’s early withdrawal from the study or a participant’s inability to evaluate an endpoint at a particular time point. The proportion of missing diary entries per participant during each reference period was classified into the following categories: 0%, > 0–5%, > 5–10%, > 10–20% and > 20%.

A missing episode was defined as one or more consecutive days with missing diary entries. The length of a missing episode was derived from the number of missing diary entries within the missing episode, and was reported for the whole observation period and by each reference period. The assignment of a missing episode to a reference period was based on the start date of the episode. Only complete reference periods were considered, which were defined as those with at least one valid entry available beyond the end of the period.

### Analyses

The mean score for worst pelvic pain for each missing episode was calculated using the worst pelvic pain scores entered directly before and directly after the missing episode. For each participant, the mean of these means was calculated. The difference between this number and the mean score of all complete episodes was also calculated for each participant and tabulated descriptively.

The proportion of missing diary entries for each day of the week was calculated, and analyses were performed for subpopulations defined by country, educational status and number of births. The Chi-squared test was used to assess the statistical significance of the difference between the proportion of missing values on Fridays or Saturdays and other weekdays, and of the difference between the proportion of patients with missing episodes longer than 1 day in the USA and Germany.

A comparison-wise significance level of 0.05 was used for this exploratory study. Statistical analyses were performed using Statistical Analysis Software (SAS) version 9.2 (SAS Institute Inc., Cary, NC, USA).

## Results

### Characteristics of the study population

The first visit of the first patient was on 31 August 2012 and the last visit of the last patient was on 29 July 2013. The mean age of the women included in the study was 31.6 years (range 19–45 years), and most participants were Caucasian (89.9% [241/268]) (Table 1). At screening, the mean score on the 0–10 NRS for pelvic pain experienced during the previous 4 weeks was 5.9 (range 0–10). As only women who had had a change in their endometriosis management participated in the study from week 12 to week 24, the number of patients per reference period reduced from 262 in reference period 1 (days 1–28) to 12 in reference period 6 (days 141–168) (Table 2).

**Table 1:** Demographic and clinical characteristics of the VALEPRO study population

Characteristic	Study population (*n* = 268)
Age, years, mean (range)	31.6 (19–45)
Race	
Caucasian	241 (89.9)
Black	22 (8.2)
Other/missing	5 (1.9)
Country of recruitment	
USA	133 (49.6)
Germany	135 (50.4)
Education level	
Elementary education	16 (6.0)
Secondary education	111 (41.4)
College or university education	113 (42.2)
Missing	28 (10.4)
Number of births, mean (range)	0.60 (0–5)
Pelvic pain score at screening^a^	
No pain (0)	1 (0.4)
Mild (1–4)	67 (25.0)
Moderate (5–6)	77 (28.7)
Severe (7–10)	107 (39.9)
Missing	16 (6.0)

Data are presented as n (%) unless otherwise stated. Of the 272 participants, full data sets from 268 women were analysed in the VALEPRO study: four participants were excluded from the analysis population owing to insufficient information across a number of variables.

^a^Self-assessment of endometriosis-associated pain at its worst in the past 4 weeks (using 0–10 numeric rating scale, where 0 = no pain and 10 = pain as bad as you can imagine). *VALEPRO* Validation Study for Endometriosis Patient-Reported Outcome

**Table 2:** Number of participants per reference period in VALEPRO

Reference period	Number of participants
1 (days 1–28)	262
2 (days 29–56)	257
3 (days 57–84)	236
4 (days 85–112)	59
5 (days 113–140)	28
6 (days 141–168)	12

*VALEPRO* Validation Study for Endometriosis Patient-Reported Outcome

### Missing data

**Fig. 2:**
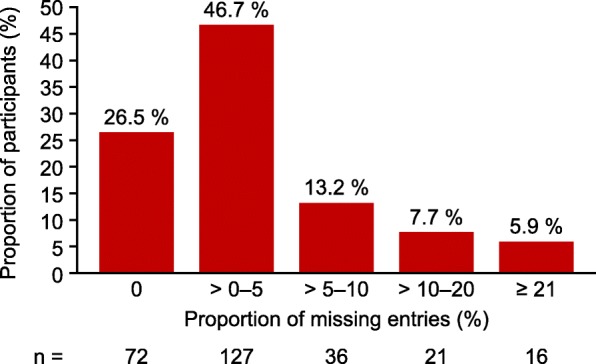
Proportion of missing diary entries per participant over the whole VALEPRO study period (*n* = 272), *VALEPRO* Validation Study for Endometriosis Patient-Reported Outcome

Most participants had high compliance with completion of the ESD, and the proportion of missing diary entries per participant was relatively low (Fig. 2). Of 272 participants, 26.5% (72/272) had no missing diary entries, 46.7% (127/272) had up to 5% of entries missing, 13.2% (36/272) had 6–10% of entries missing and 13.6% (37/272) had more than 10% of entries missing over the entire study period. The proportion of participants with missing diary entries per reference period increased over time. In reference period 1, 41.2% (108/262) of participants had at least one missing entry compared with 58.3% (7/12) in reference period 6.

Throughout the study, the duration of most missing episodes was short: 81.4% (664/816) of missing episodes were 1 day, 9.6% (78/816) of missing episodes were 2 consecutive days and 5.6% (46/816) of missing episodes were 4 or more consecutive days.

### Patterns of missing data

The scores for worst pain were very similar before and after a missing episode. The difference in mean scores between missing and complete episodes per participant was − 0.1, suggesting that missing episodes were not related to severity of pain.

**Fig. 3:**
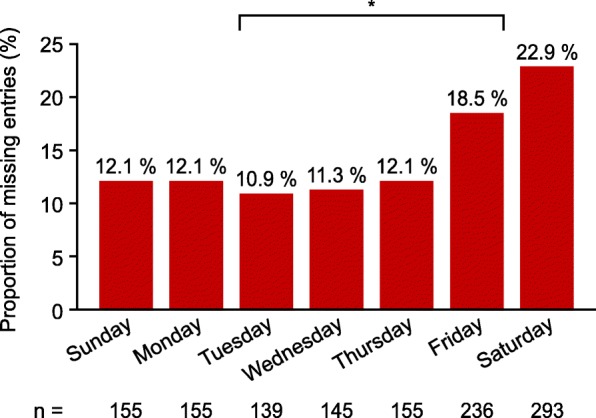
Number of missing diary entries by day of the week during VALEPRO (*n* = 1278), *p < 0.0001 between Friday–Saturday and Sunday–Thursday. *VALEPRO* Validation Study for Endometriosis Patient-Reported Outcome

Analysis of missing data by the day of the week demonstrated that entries were significantly more likely to be missing on Fridays (18.5% [236/1278]) and Saturdays (22.9% [293/1278]) than on other days of the week (Fig. 3; *p* < 0.0001). This pattern was consistent across both countries.

### Subpopulation analyses

Analysis of missing data by country revealed that participants in the USA tended to be less compliant with completing the ESD than those in Germany (proportions of women with complete diary entries, 17.6% [24/136 of participants in the USA] and 35.3% [48/136 partipants in Germany], respectively). In addition, participants in the USA had significantly more missing episodes lasting longer than 1 day than those in Germany (22.6% [124/549 missing episodes observed in the USA] and 10.5% [28/267 missing episodes observed in Germany], respectively; *p* < 0.0001).

Analysis of missing data by education status demonstrated that the proportions of women with at least one missing diary entry were 50.0% (8/16), 70.2% (80/114) and 79.8% (91/114) for women with elementary education, secondary education, and a college or university education, respectively. The proportion of women with at least one missing entry was similar for those with and without children (72.2% [70/97] and 74.3% [130/175], respectively).

## Discussion

This post hoc analysis shows that most participants were highly compliant with entering data in the ESD and that the amount of missing data was low. This supports the conclusion of VALEPRO that the ESD is reliable and valid [15].

Missing data are commonly encountered in quantitative research [17]. They can reduce the statistical power of a study, limit the representativeness of the sample, and result in biased estimates and invalid conclusions [18]. ePRO instruments can potentially reduce the amount of missing data compared with traditional paper PRO instruments by making regular data entry simpler and quicker, increasing the accuracy and integrity of the data collected. In addition, ePRO instruments can employ various techniques to reduce further the occurrence of missing data. For example, the technical design of the electronic ESD does not permit missing data at the item level because respondents are required to answer all questions on any given day. In contrast, the Endometriosis Health Profile-30 (EHP-30) [11] allows missing data at the item level, and a cross-sectional postal survey has reported a missing response rate of 0.2–1.3% for the core questionnaire [10]. Secondly, the ESD version 4.0 only has 12 questions, so it is less burdensome to complete than other endometriosis-specific questionnaires such as the EHP-30 and the Endometriosis Pain and Bleeding Diary, which consist of 30 questions and 17 questions, respectively [11, 19]. While questionnaires are not routinely used in clinical practice, it is essential to understand and minimize missing data in research studies to avoid reaching incorrect conclusions due to biases introduced by missing data. Many regulatory agencies, including the FDA and the European Medicines Agency, have published guidelines highlighting the importance of reducing the amount of missing data, and of handling missing data appropriately [12, 20]. Information on source and procedures for avoiding missing data are of major importance for informing FDA decisions on appropriateness of use of a PRO measure to support regulatory label claims [12].

In VALEPRO, a low proportion of data entries in the ESD was missing, and episodes of missing data were generally short in duration. In terms of limitations, VALEPRO was an exploratory study that only included patients from the USA and Germany. In addition, because the study was non-interventional, the number of patients experiencing a change in the severity of endometriosis during the course of the study was small. In future studies, additional information about missing data could be obtained by investigators contacting participants with missing diary entries to request the reasons for them.

Analyses assessing how missing data were related to pain severity, country and day of the week allowed assumptions to be made on the causes of missing data. The mean scores for the worst pain were similar before and after missing episodes, suggesting that there is no link between the severity of pain and the reasons for missing data. Based on the selection criteria, patients included in this study should have been physically able to complete the diary on a daily basis. However, we acknowledge the possibility that patients may have been so severely affected by pelvic pain on a given day that they were unable to complete the diary, and that the worst pelvic pain scores entered directly before and directly after the missing episode may not fully reflect the pain experienced during that missing episode.

In general, participants in the USA were less compliant with the ESD and had significantly more long missing episodes (i.e. over 1 day) than participants in Germany, which can potentially be explained by cultural differences. The other possible influencing factors that were examined, such as educational level and parity, appeared to have limited impact on compliance. Women were instructed to complete the ESD every evening via an electronic hand-held device supplied by the study sponsor. Entries were significantly more likely to be missing on Fridays and Saturdays than on other days of the week, suggesting that missing diary entries may have occurred when women went out for the evening without the device. In the future, it may be possible to reduce this potential cause of missing data by providing the ESD as an app that women can download onto their own mobile device, which they are more likely to carry with them at all times.

Patterns of missing data in the ESD are not comparable with those from other questionnaires, due to the structure of the ESD, which is programmed such that either all questions are answered or no questions are answered on a given day.

## Conclusions

The results of this post hoc analysis show that compliance with data entry in the ESD is high and the patterns of missing data do not suggest any bias associated with completion of the ESD. These results support the use of this ePRO in clinical trials assessing the impact of endometriosis-related symptoms.

## Abbreviations

EHP-30: Endometriosis Health Profile-30
EIS: Endometriosis impact scale
ePRO: electronic patient-reported outcome
ESD: Endometriosis symptom diary
FDA: Food and Drug Administration
NRS: Numeric rating scale
PRO: Patient-reported outcome
VALEPRO: Validation study for endometriosis patient-reported outcome
